# Dynamic Alignment Models for Neural Coding

**DOI:** 10.1371/journal.pcbi.1003508

**Published:** 2014-03-13

**Authors:** Sepp Kollmorgen, Richard H. R. Hahnloser

**Affiliations:** Institute of Neuroinformatics, University of Zurich/ETH Zurich, Zurich, Switzerland; Indiana University, United States of America

## Abstract

Recently, there have been remarkable advances in modeling the relationships between the sensory environment, neuronal responses, and behavior. However, most models cannot encompass variable stimulus-response relationships such as varying response latencies and state or context dependence of the neural code. Here, we consider response modeling as a dynamic alignment problem and model stimulus and response jointly by a mixed pair hidden Markov model (MPH). In MPHs, multiple stimulus-response relationships (e.g., receptive fields) are represented by different states or groups of states in a Markov chain. Each stimulus-response relationship features temporal flexibility, allowing modeling of variable response latencies, including noisy ones. We derive algorithms for learning of MPH parameters and for inference of spike response probabilities. We show that some linear-nonlinear Poisson cascade (LNP) models are a special case of MPHs. We demonstrate the efficiency and usefulness of MPHs in simulations of both jittered and switching spike responses to white noise and natural stimuli. Furthermore, we apply MPHs to extracellular single and multi-unit data recorded in cortical brain areas of singing birds to showcase a novel method for estimating response lag distributions. MPHs allow simultaneous estimation of receptive fields, latency statistics, and hidden state dynamics and so can help to uncover complex stimulus response relationships that are subject to variable timing and involve diverse neural codes.

## Author Summary

The brain computes using electrical discharges of nerve cells, so called spikes. Specific sensory stimuli, for instance, tones, often lead to specific spiking patterns. The same is true for behavior: specific motor actions are generated by specific spiking patterns. The relationship between neural activity and stimuli or motor actions can be difficult to infer, because of dynamic dependencies and hidden nonlinearities. For instance, in a freely behaving animal a neuron could exhibit variable levels of sensory and motor involvements depending on the state of the animal and on current motor plans—a situation that cannot be accounted for by many existing models. Here we present a new type of model that is specifically designed to cope with such changing regularities. We outline the mathematical framework and show, through computer simulations and application to recorded neural data, how MPHs can advance our understanding of stimulus-response relationships.

## Introduction

Neural response models are used to relate neural activity to sensory stimuli and motor behavior. A very common type of neural response model is comprised of a linear stage, at which one or more linear filters (often referred to as receptive fields) are applied to the stimulus, and a subsequent non-linear stage that converts the filter outputs into a spiking probability that feeds into a Poisson process generating the spikes [1]. More precisely, the spiking probability (i.e., the instantaneous rate of the Poisson process) of a neuron is modeled as 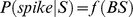, where the column vector 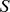 represents the stimulus, 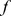 the nonlinearity, and 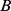 is a row vector containing the linear filter or a matrix in case of several filters. Variations of these linear-nonlinear Poisson cascade models (*LNP models*) have been studied extensively [2]–[5]. Parameter estimation techniques range from spike triggered averaging in case of one linear filter and white noise stimuli, to spike triggered covariance [1], [6] in case of several linear filters, and maximally informative dimensions, in case of one or several linear filters and no restrictions on the distribution of stimuli [2], [4]. Although these techniques are effective in many domains, they fail in others, where the neural code might be more intricate (detailed below).

**Figure 1:**
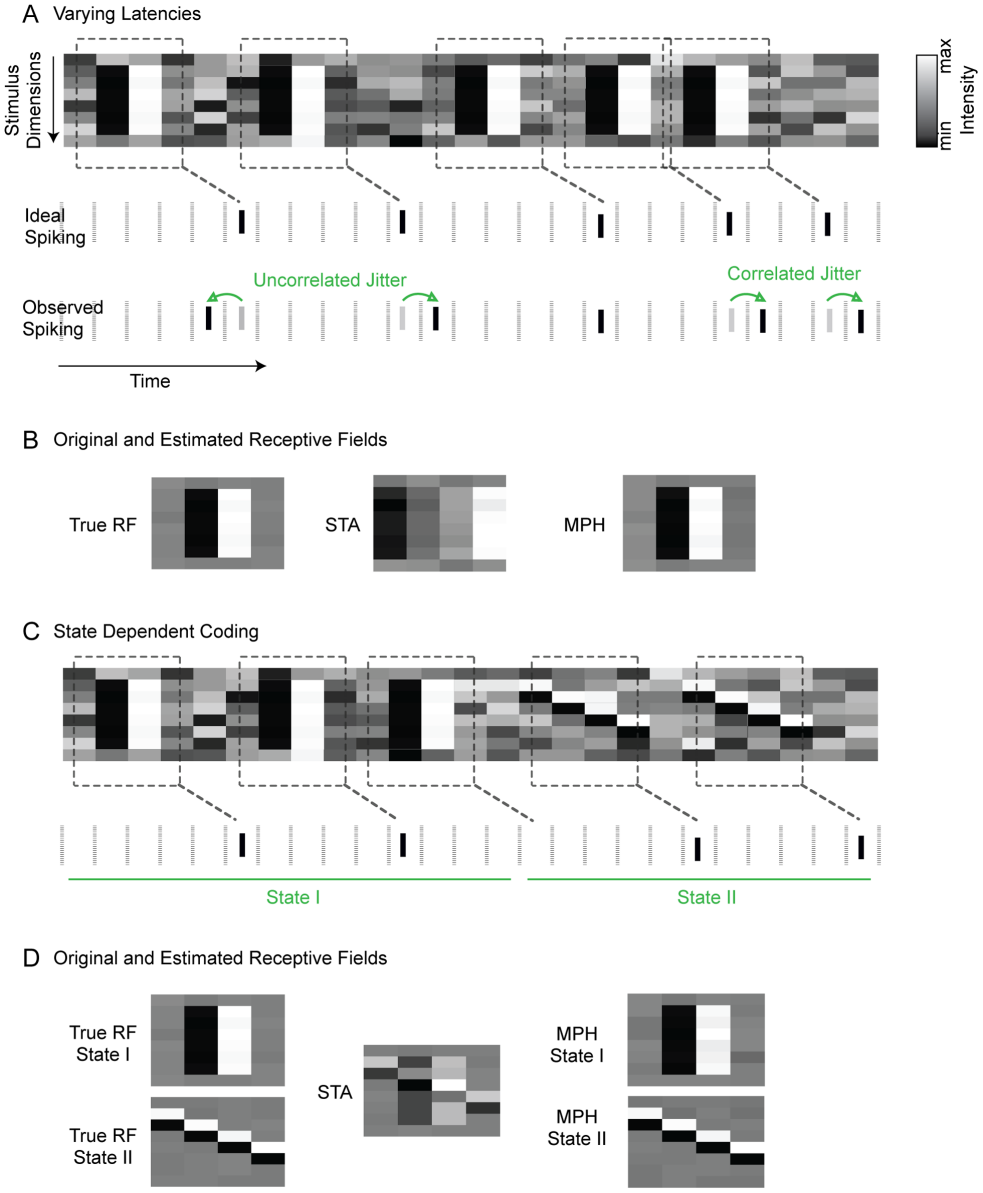
Varying response latencies and context dependent neural coding. (A) *Varying latencies*. Sequence of 8 dimensional white noise stimuli (e.g. successive frames on a one dimensional screen with 8 pixels). An LNP model generates spikes (black bars) if a chunk of stimulus (dashed rectangles) is similar enough to its receptive field (dashed rectangles). Jitter-free or ideal spikes (vertical black bars, ‘ideal spiking’) are produced with some fixed latency (dashed diagonal lines). Jittered spikes (black bars, ‘observed spiking’) are produced by randomly jittering ideal spikes (gray bars) forward or backward in time (green arrows). The jitter of adjacent spikes can be independent or correlated. The jittered spikes are the basis for fitting neural response models. (B) Receptive field (RF) estimates using spike triggered stimulus averaging (STA) on unjittered spikes (true RF), jittered spikes (STA), and the MPH on jittered spikes (MPH). Noisy response latencies lead to blurring of STA RFs, but not of MPH RFs. (C) *State-dependent coding*. For the same white noise stimulus, spikes are generated from one of two LNP models depending on hidden states I and II (green lines) determining which model is used. (D) The true RFs are superimposed when estimated with STA. A two-states MPH can faithfully recover the two RFs.

A crucial assumption about the relationship between stimulus and response inherent in these techniques is that the response latency of the cell, the filters (or receptive fields), and the non-linearity all are assumed to be the same throughout the experiment (Fig. 1A). This assumption of a *fixed stimulus-response relationship* is, however, not necessarily valid. On the one hand, the relationship between stimulus and response, the neural code, could vary in time (Fig. 1C). On the other hand, the response latency could be noisy or vary systematically (Fig. 1A).

The extent to which a *fixed stimulus-response relationship* applies to neurophysiological data is unclear. In terms of spike timing, a fixed relationship entails both constant response latency and an amount of spike-time-jitter that is smaller than the relevant temporal structure of the receptive field. However, noisy response latencies (Fig. 1A) are observed in almost all electrophysiological studies because neural systems are intrinsically noisy. Variations in response latency to a repeated stimulus (measured as the standard deviation of time of first spike after stimulus onset) in the range of 3–5 ms have been reported already at a very low stage of the visual system, in retinal ganglion cells [7]. Variability in response latency can be notably larger in cortical areas. For instance, variations in first-spike latency (again measured as the standard deviation) of up to 12.5 ms have been observed in single cells in ferret primary visual cortex in response to flashed natural images [8]. Furthermore, systematically varying response latencies have been demonstrated in various model systems, e.g., image contrast modulates response latency both in retinal ganglion cells [9] and in visual cortical neurons [10]–[12], fueling discussions about the role of spike latency in neural coding [12], [13]. In a recent study, latencies of cells in macaque inferotemporal cortex were found to systematically differ for primate and non-primate face stimuli, with latency differences on the order of tens of milliseconds [14].

When latencies are strongly fluctuating or spike time-jitter is large, many modeling techniques that assume a *fixed stimulus-response relationship*, such as spike triggered averaging, yield suboptimal results [15], [16]: The estimated receptive fields are blurred and the accuracy of predicted responses to novel stimuli is low (Fig. 1B).

*Fixed stimulus-response relationships* can also be violated in case of changes in intrinsic or hidden brain states [17], [18]. For example, neurons in the primary somatosensory cortex of the rat undergo up and down states given by two separate membrane potentials. Spiking responses to whisker deflections in these cells are dependent on whether neurons are in the up or the down state: in the down state, a reliable response is observed, whereas in the up state activity is largely stimulus independent [19]. Stimulus context can also induce changes in internal states. For instance, in an awake marmoset study of single-unit responses in the auditory cortex to sequences of 2 sound stimuli, responses to the second stimulus were not static but depended strongly on the first stimulus. This modulation in second-stimulus responses can last longer than 1.5 seconds [20]. We illustrate dependence of neural computation on intrinsic states in a cartoon (Fig. 1C) in which the simulated neuron switches between 2 static receptive fields. Response switching has also been observed in the amphibian retina. Ganglion-cell activity is typically dominated by OFF responses. However, a large peripheral image shift (as occurs during head saccades) can induce a switch (for a few hundred milliseconds) from transmitting OFF signals to transmitting ON signals [21]. One of the most compelling examples of response switching has been observed in songbirds: many neurons in cortical motor and auditory areas are responsive to playback of sound stimuli except when birds are singing, at which times responses are locked to the song but not influenced by sound playback [22], [23]. Hence, if such neural responses were to be modeled across singing and non-singing states, anything but a two-state model would be inadequate. Indeed, many classical models fail in cases of response switching: the estimated classical receptive fields contain superimposed structures derived from the switched responses, which yields sub-optimal results (Fig. 1D).

To address variable response latencies and dynamic neural codes we consider the problem of neural response modeling as an alignment problem. We introduce mixed pair hidden Markov models (MPHs) as novel neural response models allowing for dynamic alignment of stimulus and response without *fixed stimulus-response assumptions*. In the case of varying spike latencies (e.g. when a neuron fires in response to a particular stimulus but with a variable latency or lag, Fig. 1A, B), MPHs help to detect corresponding stimulus-response parts by associating individual spikes with particular stimulus time points; and, they help to uncover stimulus-response relationships including the spike-jitter statistics and the receptive field of the neuron. In case of switching dynamics (e.g., when a neuron switches between being responsive to either one stimulus or another depending on the behavioral state of the animal or a cueing stimulus, Fig. 1C), MPHs help to identify parameters such as the receptive-field switching probabilities and the switching events. Our MPH approach to dynamic alignment combines response switching (*context dependency*) and spike-time jitter or systematically varying latencies (*flexible timing*) in one unified framework. We show how to use stimuli and neural responses to jointly estimate all model parameters including spike time jitter, systematically varying latencies, and switching probabilities. We demonstrate the benefits of dynamic alignment on simulated data and on extracellular data recorded in cortical brain areas of singing birds.

## Results

### Mixed Pair Hidden Markov Models

We solve the alignment problem by jointly modeling stimulus and response by a mixed pair hidden Markov model (MPH), which is a generative model for both the stimulus and neural response. In MPHs, different neural codes, i.e. different relationships between neural activity and sensory input, can coexist as different states or groups of states in a Markov chain. MPHs are unlike classical hidden Markov models because they dynamically operate on pairs of sequences - neural activity and stimulus - instead of single sequences (i.e. a joint sequence of neural activity and stimulus). For a mathematically detailed introduction to MPHs (and an introduction to HMMs), see the Materials and Methods section.

**Figure 2:**
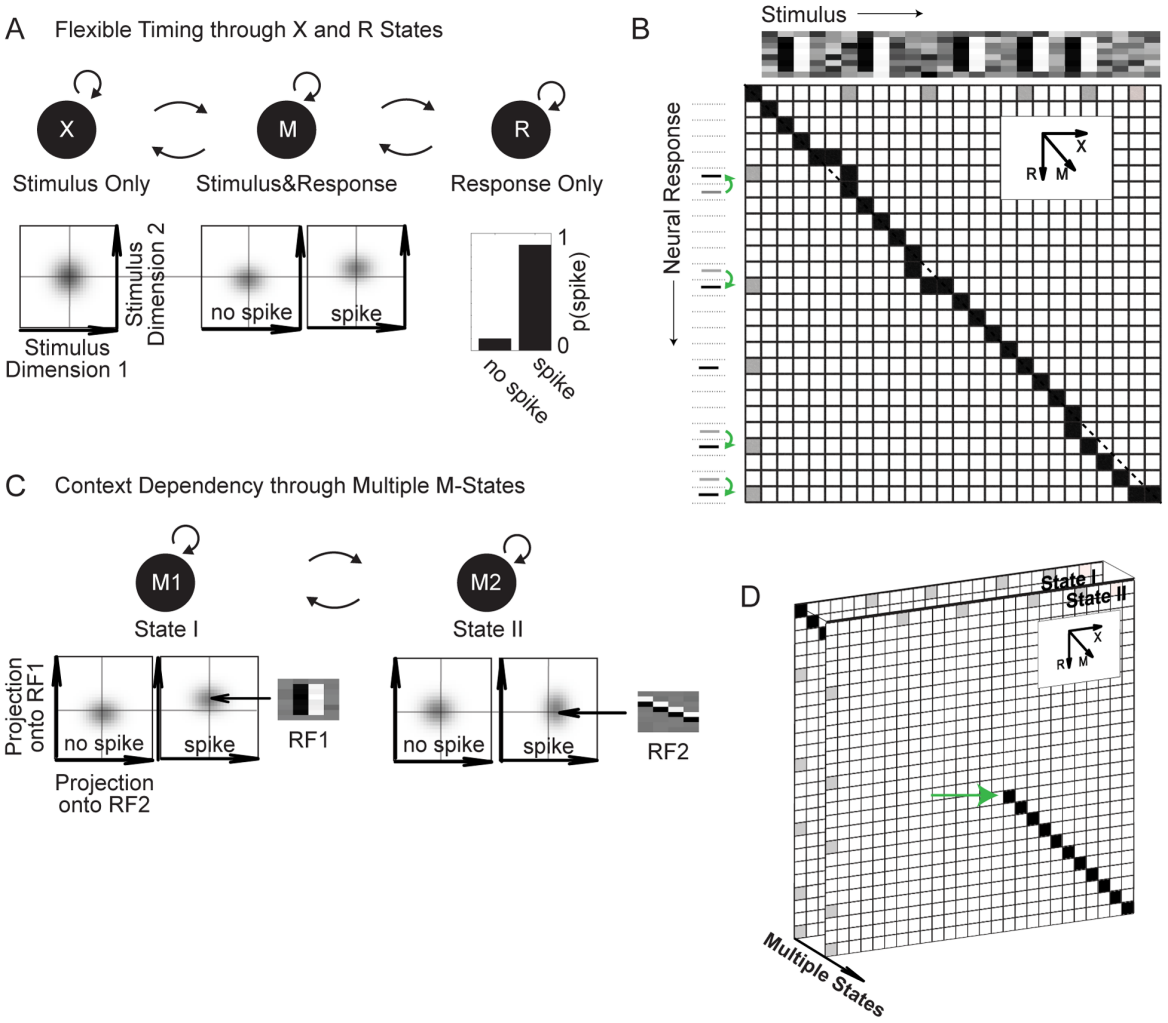
Two minimal MPHs for flexible timing and context dependent coding. (A) Architecture of the minimal MPH that allows for neural codes with varying latencies, i.e. flexible timing. This MPH has 3 hidden states, one X-state that models only the stimulus, one R-state that models only the neural response, and one M-state that jointly models stimulus and response. The probability distributions over stimuli (bottom) are illustrated as low dimensional projections (stimulus dimension 2 coincides with the receptive field of the M-state). (B) Hidden state sequences in that model correspond to paths in the alignment matrix: a diagonal step leading into position 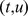 implies that stimulus and response at times 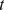 and 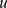 are jointly modeled by an M-state, a horizontal step implies modeling of only the stimulus at time 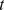, and a vertical step implies modeling of only the response at time 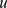 (deviations from the diagonal reflect jittered spikes detected by the model). Depicted stimulus and spiking responses are from figure 1A. (C) The minimal MPH for modeling state-dependent neural codes. The MPH can switch between several M-states, each of which represents a different RF. The (projected) stimulus distributions given a spike (spike triggered stimulus ensemble) are centered on the respective RFs (indicated by black arrows). (D) Adding states to the model turns the alignment matrix into an alignment tensor composed of several planes (strictly speaking, B depicts a tensor as well; we just projected all the states onto one plane). The switch from state 1 to State 2 is indicated (green arrow).

We explain the workings of MPHs in intuitive terms by considering first the special case of jittered spike times (Fig. 1A). We assume spikes are associated with the stimulus (i.e. a time window of the stimulus [1]) that precede the spikes by an average time lag 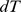. Instead of associating spikes and stimuli at a constant lag 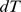 (such as is the case for standard spike triggered methods including STA, STC, maximally informative dimensions, etc..), MPHs associate an individual spike with a stimulus at the individual lag 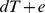 (in units of stimulus-response bins, 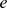 being an integer, 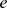 can be different for each spike). MPHs achieve this flexibility via three different types of hidden states and by keeping track of the momentary lag 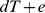 and its evolution. First, *matching states (M-states)* associate a spike with a stimulus at the current lag 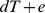 by modeling the joint probability distribution of spike and stimulus (Fig. 2A, middle). The simplest case are Gaussian stimulus models comprising two Gaussians, one of which models stimuli jointly occurring with spikes and the other models stimuli not occurring with spikes (Fig. 2A, middle). A model with only a single such M-state is bound to a fixed lag 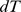 and is equivalent to an LNP model (under appropriate parameter constraints, see also the section on LNP equivalence below). To achieve a variable lag, we introduce two more types of states: X-states (X stands for the stimulus sequence) and R-states (R stands for response sequence). These states can change the momentary lag 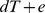 as follows. An X-state models the stimulus (but not the response) via some probability distribution, for instance a Gaussian (Fig. 2A, left). The X-state changes the current lag from 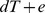 to 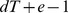. Analogously, an R-state models only the spiking response (but not the stimulus) via a discrete probability distribution (e.g. spike or no-spike, Fig. 2A, right). An R-state changes the current lag from 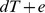 to 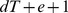. An MPH consisting of an M, X, and R state can thus model a spike-stimulus pair via the M state or via the X and the R state. In general fewer states are preferred as there is a cost associated with switching from one state to another. Thus, an MPH will try to keep the lag between stimulus and spike constant unless there is evidence for changing the lag via X and R states.

Intuitively, one can think of an MPH as a finite state automaton that processes symbols from two sequences at the same time, the stimulus (X-sequence) and the response sequence (R-sequence). Under this analogy, M-states process one symbol of each sequence (they match a symbol pair from X and R), X-states process only a stimulus symbol, and R-states process only a response-symbol. The automaton keeps track of two pointers that indicate the current position in the stimulus sequence as well as the current position in the response sequence. The pointer difference corresponds to the current lag dT+e (the “automaton” considers all possible lags, weighted probabilistically).

A sequence of hidden states in an MXR-MPH (M, X, and R states) can be depicted as a path in an alignment matrix that spans all possible pairings between stimulus and response (Fig. 2B): An M-State corresponds to diagonal movement along the matrix from position 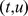, i.e. position 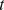 in the stimulus sequence and 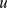 in the response sequence, to position 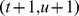. An X-State corresponds to horizontal movement from position 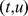 to 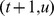 and an R-State corresponds to vertical movement from position 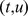 to 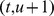. A change in the temporal relationship between stimulus and response (i.e. spike jitter) is reflected in non-diagonal (horizontal or vertical) steps in the alignment matrix (with step size provided by the discretization of stimulus and neural response sequences, Fig. 2B). This 3-state model will be applied to simulated and real data in the next section.

In order to handle state-dependent (switching) neural responses (Fig. 1C), we consider MPHs with several M-states (Fig. 2C). Multiple M states add another dimension to the alignment matrix, which we henceforth call alignment tensor (Fig. 2D). The simplest switching-enabling MPH has two M-states but no X or R states. Each M-state is associated with a particular receptive field to be estimated (here we use ‘receptive field’ in the most general way, independent of linearity and related assumptions). With several M states, spikes can be associated to stimuli via one of several joint probability distributions over spike and stimulus. Probabilistic transitions between M states allow the MPH to switch between receptive fields, which is shown on simulated and real data below.

The general MPH has several M-, X-, and R-States and thus simultaneously permits flexible timing and context dependency. The parameters defining probabilistic transitions into and out of hidden states are:

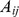
: Transition probability of transiting from hidden state 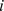 to hidden state 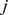,

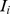
: Initial probability of hidden state 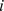,

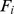
: Final probability of hidden state 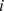.

The parameters defining emission probabilities are:

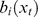
: Emission probability density of the stimulus 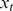 given hidden X-state 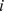 (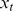 is a vector and typically spans a window of the stimulus around time *t*),

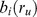
: Discrete emission probability distribution of the response 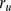 given hidden R-state 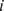 (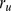 is part of a discrete set, e.g. {0, 1} for spike or no spike),

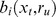
: Mixed discrete-continuous emission probability of stimulus-response pair 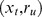 given hidden state 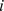

As emission probability densities associated with X and M states we use multivariate Gaussians or mixtures of Gaussians, respectively. For hidden X-state 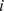 we write the emission probability density as
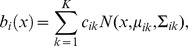
where 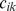 is the weight of the *k* ^th^ mixture component, 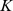 denotes the total number of mixture components (which may vary for different hidden states, i.e. some 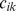 can be zero), and 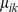 and 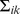 denote the Gaussian mean and covariance matrix of the *k* ^th^ mixture component. For M states we keep track of one such multivariate Gaussian for each response state 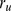 (each response state is associated with a distinct stimulus emission).

### Special Cases of MPHs

In the following we present detailed analyses of the spike-jitter and response-switching MPH architectures. First, we discuss an MPH with only one M-state (*M-MPH*) and multivariate Gaussian stimulus models. We show that, under appropriate parameter constraints, such a model is equivalent to a 1-dimensional LNP model and describe its relation to linear regression and linear discriminant analysis and the resulting strengths and limitations. Second, we discuss an extension of that M-MPH to a model that also possesses an X- and an R-state (*MXR-MPH*). We illustrate in simulations how this model can account for spike time jitter and varying latencies on white noise and on natural stimuli. Third, we treat an extension of the M-MPH to multiple M-states (*M^n^-MPH*) and illustrate through simulations how this model can account for switching dynamics and context dependency. All of these models can be cascaded with an additional non-linearity so that they form NNP cascades (as opposed to LNP cascades). In the chapter that then follows, we apply these models to data recorded from single units of cells in the behaving bird.

#### The M-MPH with multivariate Gaussian stimulus models and equal covariances

An MPH characterized by one M-state, multivariate Gaussian stimulus models with shared covariance matrix, and no X and R states is equivalent to an LNP model. In the following we calculate both the linear filter and the LNP non-linearity. Let 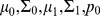, and 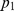 be the parameters of an MPH with one M-state and Gaussian stimulus models, where 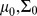 are the mean and the covariance matrix of the stimulus given no spike is emitted and 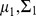 are the mean and the covariance matrix of the stimulus given emission of a spike. In this section, we assume identical covariance matrices: 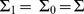. 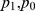 are the marginal (or prior) probabilities of a spike and no spike and 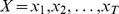, 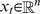 denotes the stimulus sequence and 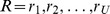, 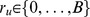 the response sequence. In the examples to follow, stimulus and response have the same length, 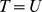 (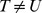 can be useful too, for instance, when stimulus and spiking response are differently binned).

In this simple MPH the posterior probability of a spike at time 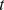 is given by
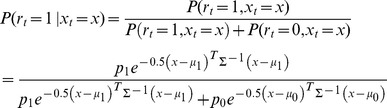
(1)


By using 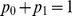 and rearranging terms, we can transform Eq. 1 to
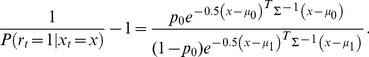
(2)


By taking the logarithm on both sides of Eq. 2 we find
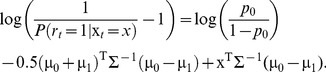
(3)


Hence, 
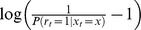
 is affine-linear in 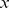. Equivalence between this simple MPH and linear non-linear neural response models follows after applying the sigmoid function, 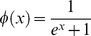, on both sides of (3) and assuming 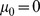, which yields
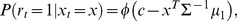
(4)where the constant 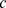 is given by 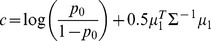. Hence, the posterior spike probability 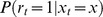 for this MPH agrees with that of an LNP model with linear filter (or receptive field) 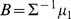 and non-linearity
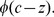
(5)


For non-white stimuli, i.e. 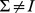 with 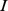 the identity matrix, the receptive field 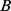 of the MPH is given by
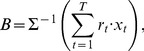
(6)which is known as reverse correlation, corrected spike triggered average [24], [25], or simply as linear regression. Moreover, for white stimuli, i.e. 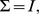 the receptive field 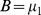 of the MPH is the spike triggered average (STA) - the mean of the spike triggered ensemble. Hence, the M-MPH performs a linear regression cascaded with a sigmoid non-linearity and is equivalent to a one dimensional LNP model with a sigmoid non-linearity (Eq. 5).

Note that the M-MPH's receptive field (Eq. 6) always corresponds to the linear regression solution. Consequently, the M-MPH's receptive field estimate is optimal whenever linear regression is the correct model. In particular, it follows that the MPH parameter estimates can be optimal even when the spike triggered ensemble and its complement are non-Gaussian – for instance in case of white noise stimuli and a threshold non-linearity [1], [26]. The same is true when the overall stimulus distribution is non Gaussian – for instance for non-Gaussian natural stimuli and a linear “non-linearity” [26]. However, although the receptive field estimate does not depend on Gaussian assumptions, the non-linearity (Eq. 5) does, see e.g. [26]. In [26], the authors suggest to re-estimate the non-linearity (decision boundary) of a linear discriminant model to obtain a non-linearity estimate not corrupted by Gaussian assumptions.

Inspired by [26], we cascade the MPH with an additional non-linearity that can be estimated from the data subsequent to the estimation of the MPH (compare Materials and Methods for details of the estimation). Such cascading is standard practice for neural response models [1], [27], [28] and is also part of LNP models. The cascaded M-MPH with shared covariance can fit any one-dimensional LNP model on white-noise data (provided that the operation of the LNP model leads to a change of mean of the spike triggered ensemble, which is the case for all monotone non-linearities and for most others) [1]. However, for non-white data and certain nonlinearities, the linear regression estimate can be biased [1], [2], so that MPHs too will infer an incorrect receptive field (see also below, where we apply MPHs to natural stimuli). In these cases, using Gaussian mixtures as stimulus models might be advisable (see discussion).

#### M-MPH with free covariance matrices and Gaussian mixture models

One interesting extension of that simple MPH results from assuming 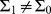. This case is analogous to quadratic discriminant analysis [26]. The MPH implements a model quadratic in 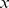. One further extension is to use mixtures of Gaussians as emission distributions instead of individual Gaussians (see discussion), in which case 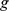 is generally not quadratic anymore.

### The MXR-MPH and Its Application to Simulated Data with Spike-Time-Jitter

In the following we demonstrate the ability of MXR-MPHs (Fig. 2A) to recover the correct receptive field (RF) on simulated data with noisy latencies, i.e. spike-time-jitter. When predicting spiking probabilities on novel data, the MXR-MPH outperforms purely spike-triggered methods.

For the MXR-MPH, we denote the stimulus means and covariance matrices in the M-State by 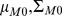 (non-spiking) and 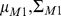
(spiking) and in the in the X-State by 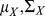. In accordance with the section on LNP equivalence and to simplify this general MPH, we introduce the following parameter constraints: First, we fix all covariance matrices to identity matrices: 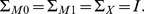



Second, we fix the means 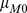 and 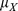 to zero (equal to the mean of the stimulus ensemble). Third, we do not allow the R-state to generate spikes (zero spike emission probability) because we require that each spike is matched to a stimulus (note that if we allowed the R-state to generate spikes, the model would distinguish between spikes generated by the M-state and spikes generated by the R-state — such distinction could be used for distinguishing stimulus driven from spontaneous activity, which was not our focus). Given these parameter constraints, the remaining free parameters in the model are the receptive field 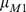 (the mean of the spike triggered stimulus ensemble, Fig. 2C), and the transition probabilities among X, R, and M-state. After training the MPH, the jitter statistics are implicit in the model's parameters; below we show how to explicitly compute the jitter statistics for natural stimuli.

#### Application to white data

**Figure 3:**
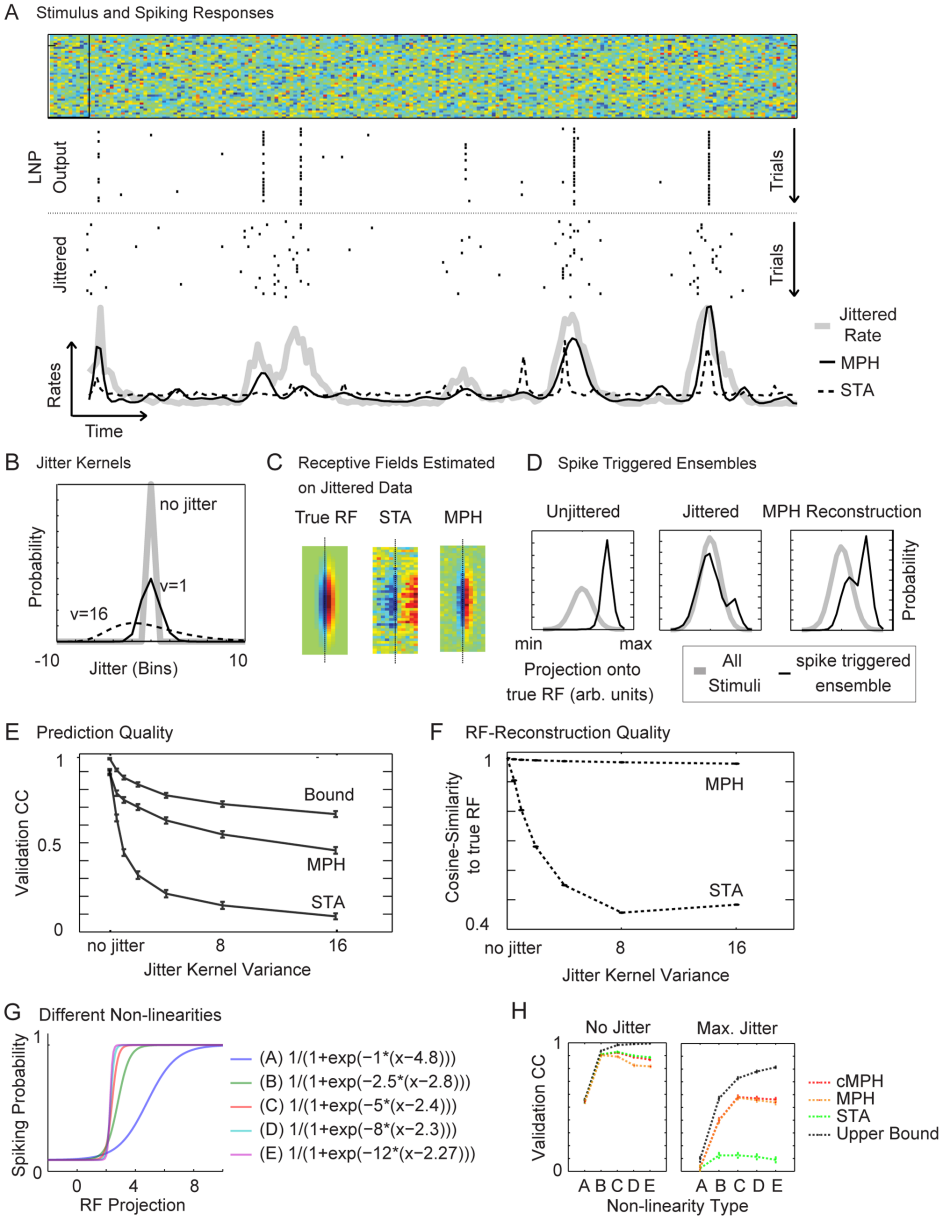
The MXR-MPH applied to white noise stimuli and spike-time-jitter. (A) A white noise stimulus (top) with spiking responses (black bars) generated by an LNP-type model neuron (LNP output, the LNP RF size is indicated by the black rectangle). The jittered versions (jittered) of the LNP spike trains with corresponding firing rate (thick gray line) are shown below. The MPH estimate of firing rate (black full line) is more accurate than the STA estimate (dashed line). (B) Applied spike jitter is i.i.d. among spikes and log-normally distributed with zero mean (3 different jitter distributions are shown; they differ in terms of variance 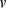 and symmetric/asymmetric shape). Results for the jitter kernel with variance 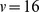 are shown in panels A, C and D. (C) RFs estimated through STA on unjittered spikes (true RF), STA on jittered spikes (STA), and MPH on jittered spikes (MPH). The STA RF is blurred whereas the MPH RF closely resembles the true RF. Dotted black lines indicate the midpoints of the RFs. (D) Projections of all stimuli (gray lines) and the spike triggered stimulus ensembles (black lines) onto the underlying (true) RF for the unjittered spikes (left), the jittered spikes (middle), and the MPH reconstruction (right, obtained via dynamic alignment using the generalized Viterbi algorithm). (E) *Response prediction*. To evaluate the models we computed correlation coefficients (CCs) between predicted and actual firing rates on the validation set and for different jitter variances. For small spike jitter, performances of STA and MPH are comparable. As the jitter magnitude increases, STA performance drops much more severely than does MPH performance. Also shown is an upper bound for the CC computed by sampling and cross-correlating jittered responses. (F) MPH robustness to jitter is demonstrated also when assessed as similarity between the estimated RF and the true RF (similarity computed as normalized scalar product, i.e. cosine of angle between RFs). (G) We assessed the influence of different non-linearities (labeled A–E, ordered by steepness) on prediction quality for both the MPH as well as the cascaded MPH (cMPH). (H) Shallow non-linearities decrease the upper bound of prediction quality (black line) as well as the MPH (red lines) and STA (green line) performance for the unjittered (left) and the jittered case (right). The cascaded MPH (red line) shows slight improvements over the non-cascaded one (dotted red line).

To test the model, we first created artificial data by sampling spike trains from an LNP model in response to a white noise stimulus consisting of 10^5^ time bins (arbitrary timescale), and 42 dimensions or stimulus channels (e.g. pixels on a one dimensional screen or frequency bands in a spectrogram). We split the stimulus into 500 sequences of equal duration and generated 25 trials of LNP spiking responses for each of those sequences (Fig. 3A). The LNP model was composed of one linear filter that spanned 11 time bins (Fig. 3C) and a sigmoid nonlinearity (red line in Fig. 3G). We generated a total of 28702 LNP spikes (∼0.015 spikes/bin). We randomly jittered the individual LNP spikes (Fig. 3A) with i.i.d. spike shifts drawn from a discretized log-normal distribution with zero mean (Fig. 3B). The variance of that distribution controls the total amount of jitter: As variance increases, the distribution becomes more asymmetric with a heavy right-side tail (we choose such an asymmetric jitter distribution to increase the difficulty of the problem). To 450 of the 500 stimulus-response sequences we fitted both an MXR-MPH and a reverse correlation model (resulting filters are depicted in Fig. 3C). The remaining 50 sequences served as validation set.

The STA, which is the optimal solution in case of jitter-free data, yielded a poor approximation of the true receptive field (Fig. 3C). The main problem for STA was that the (jittered) spike-triggered stimulus ensemble did not separate well from its complement (Fig. 3D, middle) when projected onto the underlying (true) RF. In contrast, the receptive field 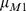 estimated using the MXR-MPH (Fig. 3C) was very close to the true RF, and the reconstructed spike-triggered ensemble (computed by aligning stimulus and response through the generalized Viterbi algorithm, see Materials and Methods) was well separated from the full stimulus ensemble (Fig. 3D, right). We ran the spiking-probability inference algorithm outlined in Materials and Methods on the independent validation set. The MPH-predicted spike responses were in general much better than STA-predicted responses (Fig. 3E, instead of using correlation coefficients, we could have evaluated performance through the average likelihood of the models given the data; we opted for CCs to ensure easy interpretability and connect to existing literature). For small jitter, MPH and STA responses were equally good; however, with increasing jitter, the MXR-MPH performance dropped much less than that of STA. Similar superiority of the MXR-MPH was also seen in RF estimation, evaluated in terms of the angle between estimated and true RFs (Fig. 3F, to discount for arbitrary shifts in RF position that could be induced by the asymmetric jitter kernels we also designed a shift-invariant measure by time-shifting the estimated RF relative to the true RF and considering only the minimal angles; this gave virtually identical results).

We elucidate the influence of various non-linearities, by having evaluated MPHs and STA-models for various sigmoidal non-linearities of the (true) LNP model (Fig. 3G, the non-linearities were chosen such that the resulting models each yield an average rate of about 0.015 spikes/bin). We found that RF estimation and response prediction of the MPH was the better the steeper the non-linearities (Fig. 3H). Furthermore, the difference between cascaded and non-cascaded MPH is not large (Fig. 3H). The improved performance for steep nonlinearities is to be expected because the non-linearity is needed to separate the action of the linear filter from the action of the jitter kernel in the underlying LNP model (i.e. without non-linearity the linear kernel of the LNP model and the jitter kernel simply act as two subsequent linear operations which no longer can be uniquely disentangled).

#### Application to natural stimuli

**Figure 4:**
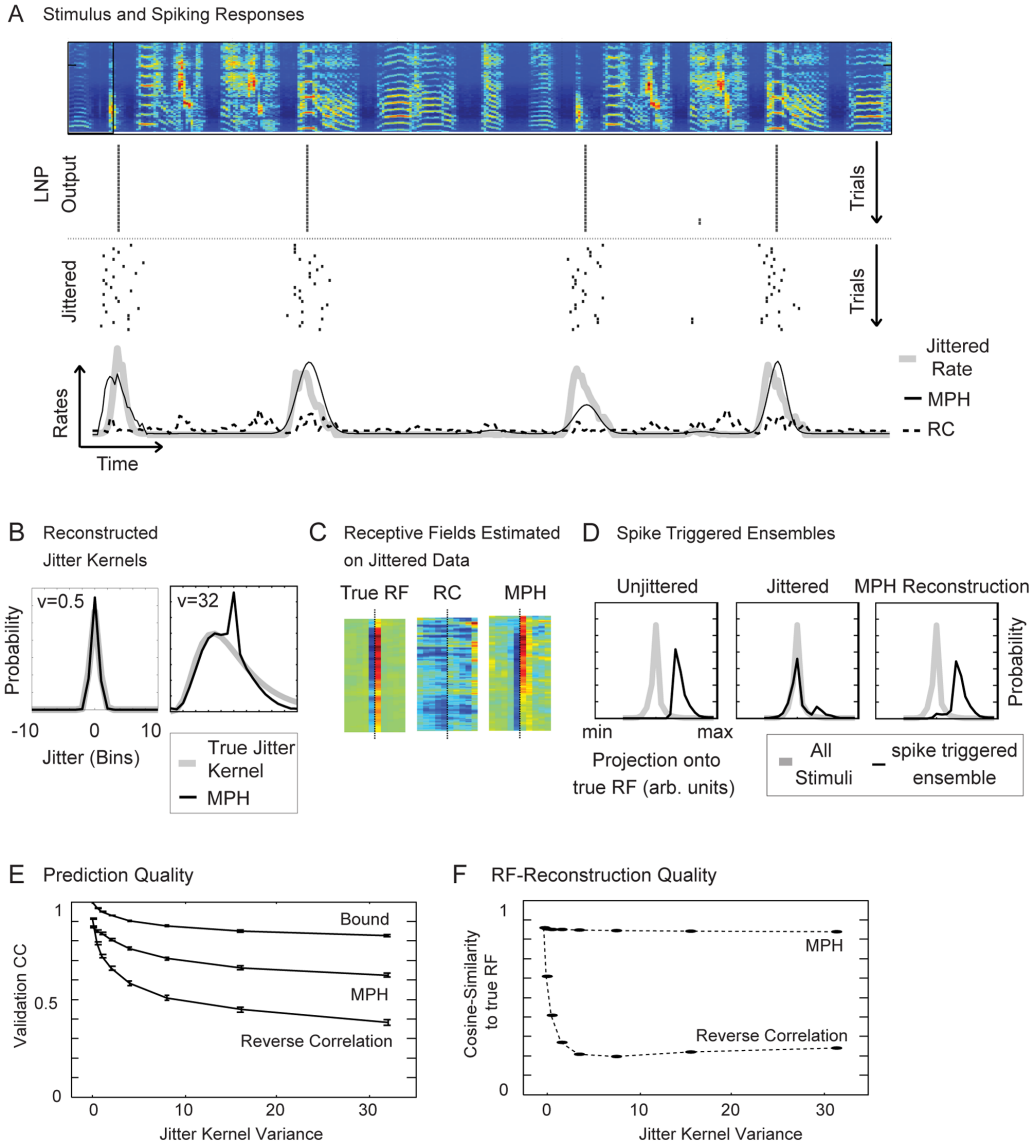
The MPH applied to natural stimuli and jittered spike responses. (A) An example log-spectrogram of zebra finch song (top, high sound amplitudes in red and low amplitudes in blue), spiking responses generated by an LNP-type model (middle, LNP output), their jittered versions (below), and the corresponding jittered firing rate (bottom, gray line). The MPH-predicted response (MPH, full line) of the jittered firing rate is more accurate than the reverse correlation prediction (RC, dashed line). (B) Applied spike jitter is i.i.d. among spikes and log-normally distributed with zero mean. Two different jitter distributions are shown, they differ in terms of variance 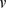 and symmetric/asymmetric shape (gray curves 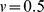 left, and 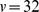 right). The MPH-estimated jitter kernels are shown in black. The MPH misses some jittered spikes (right), as revealed by the excessive peak at zero time lag. Results for the jitter kernel with variance 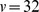 are shown in panels A, C, and D. (C) RFs estimated through reverse correlation for unjittered data (true RF), jittered data (RC) as well as the MPH receptive field estimate (MPH). The STA RF is blurred whereas the MPH RF closely resembles the true RF. Dotted black lines indicate the midpoints of the RFs. (D) Projections of all stimuli (gray lines) and the spike triggered stimulus ensembles (black lines) onto the underlying (true) RF for the unjittered spikes (left), the jittered spikes (middle), as well the MPH reconstruction (right, obtained via dynamic alignment using the generalized Viterbi algorithm). (E) Correlation coefficients (CCs) between predicted and true firing rates on the validation set for different jitter variances. Also shown is an upper bound for the CC computed by sampling and cross-correlating jittered responses. For small overall jitter, performances of reverse correlation and MPH are comparable. As the overall jitter magnitude increases, reverse correlation performance drops much more severely than does MPH performance. (F) RC performance drops even stronger when assessed in terms of similarity between the estimated and the true RFs.

We also tested the MXR-MPH on jittered responses to natural stimuli (a problem that, to our knowledge, has not yet been addressed in the literature). We sampled spike trains from an LNP model in response to spectrograms of birdsongs (Fig. 4A). We used 250 zebra finch songs (50 of the songs served as a validation set), yielding a total of 66025 time bins (4 ms each) and 20332 generated spikes (mean rate/bin ∼0.012).

We fixed the model covariance matrices to the covariance 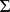 of the stimulus ensemble:
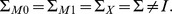



Furthermore, we fixed the means 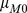 and 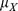 to the actual stimulus mean. As for white noise stimuli, the MPH performed better than reverse correlation on RF estimation and response prediction (Fig. 4E, 4F).

We computed the jitter statistics via the alignment kernel of the MXR-MPH (the alignment kernel is a weighted average of spike shift counts associated with each possible hidden state sequence (i.e. each path in the alignment tensor, Fig. 2B) where the weights correspond to the respective probabilities of the hidden state sequences given model and data, see Materials and Methods). Our simulations showed that the model did not over-fit the data by detecting jitter when none was present (Fig. 4B, left) and that the alignment kernel could be estimated quite well even when jitter was large (Fig. 4B, right).

The MPH allows for stimulus-response modeling both for correlated and uncorrelated jitter: Correlated jitter can be accounted for by decreasing the transition probabilities onto X and R-states, which in turn decreases the probability of non-diagonal movement in the alignment tensor (thus leading to correlated stimulus-response lags across successive spikes). To model uncorrelated jitter, the transition probabilities can be chosen such that the likelihood of a chunk of the stimulus-response pair being modeled with only X- and R-states equals the likelihood of modeling it with M-states only. In that case, constant time lags and changing time lags between successive spikes are equally likely and jitter is uncorrelated (provided that successive spikes are further apart than the jitter size).

### The M^2^-MPH and Its Application to Simulated Data with Switching Dynamics

**Figure 5:**
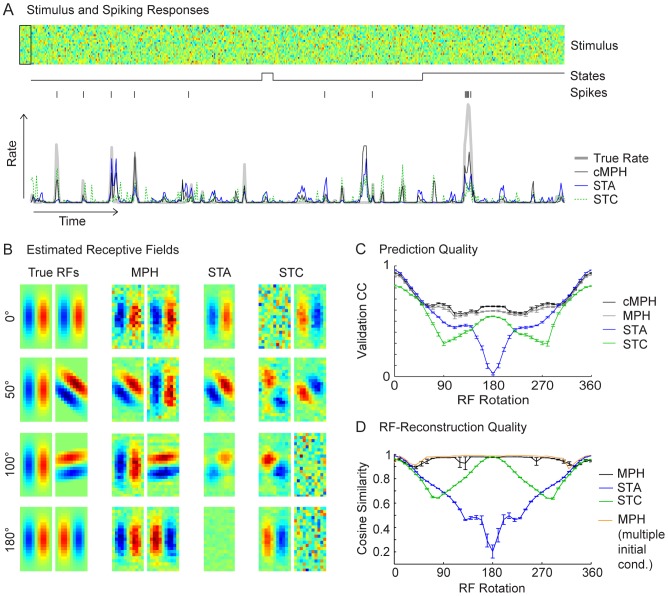
The MPH applied to white noise stimuli and switched responses. (A) A white noise stimulus (top), the randomly switched states of a switching LNP model (middle, black curve), and the observed spike train (middle, black rasters) and firing rate (bottom, gray line). The MPH-predicted firing rate (bottom, black line) to a test stimulus is closer to the observed firing rate than is the STA prediction (blue line) or the STC prediction (dotted green line). (B) The MPH RF estimates (MPH, 2^nd^ column) capture well the underlying true RFs (True RFs, 1^st^ column) for all relative angles, unlike the STA RF estimates (STA, 3^rd^ column) or the STC RF estimates (STC, 4^th^ column). (C) We evaluated the models by computing CCs between predicted and observed firing rates on a validation set and for different pairs of LNP filters that were generated by rotating one of the RFs. The cascaded MPH (black line) performs slightly better than the non-cascaded MPH (gray line). Both MPHs perform better than STC (green line) and STA (blue line). (D) Quality of RF reconstruction, shown is the cosine angle between true and model RFs (compare main text). The MPH reconstructed the true RFs more faithfully (black line) than did STA (blue line) and STC (green line). The occasional drops in MPH performance (larger error bars) are due to local optima that can be circumvented by starting the MPH-parameter optimization from different initial conditions (the orange line is from the best model – in terms of likelihood on the training set – out of 3 initial conditions). Both, panels (C) and (D) show average results from 10 simulations (with standard errors indicated).

MPHs with several M states support *context dependency*. They can model multiple stimulus-response relationships associated, for instance, with distinct behavioral states of an animal. To demonstrate this flexibility of MPHs, we simulated a neuron that randomly switches (according to a Markov process with equal probabilities) between two linear-nonlinear models (each defined as in the previous section), i.e., neural responses were governed by a hidden state sequence that determined which receptive field was active at any given time (Fig. 5A). We generated responses of this artificial neuron to 100 white noise sequences, each spanning 1000 time bins (arbitrary timescale), and 21 dimensions or stimulus channels. For each sequence, spike responses were generated using the switching LNP model, resulting in a total of 4374 spikes on average (mean 0.044 spikes/bin). We generated and tested data for different RF combinations, characterized by different rotations in the plane of one of the RFs (Fig. 5B, left). To uncover the hidden switching dynamics and the two RFs, we trained a Gaussian M^2^-MPH with two M-states 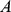 and 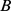 (Fig. 2C). Gaussian parameters were constrained in the following way: First, we imposed zero means: 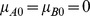; and second, we fixed all covariance matrices to the identity matrix. The remaining free parameters of the model were the means 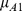 and 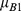 of the spike-triggered ensembles (i.e., the receptive fields) and the transition probabilities between the two states (which reflect the switching statistics).

We compared the MPH with STA and STC [1], [6] models. For all three models, we computed RF estimates and the response prediction performance. The trained MPH faithfully recovered both RFs, whereas the (single) RF estimated of STA consisted of a superposition of the two RFs, and the RFs estimated using STC were severely corrupted by noise in most cases (Fig. 5B). As a result, the MPH predicted responses better on an independent validation set than did STA and STC (Fig. 5C, averaged over 10 runs). We assessed the quality of the recovered RFs of all three methods and all 37 tested rotations by matching each original RF to the recovered RF with smallest distance and by averaging the two distances. The MPH recovers the RF much better then STA or STC (Fig. 5D, average over 10 runs; drops in MPH-reconstruction quality are due to local minima, compare figure text).

The degraded performance of the STC model has two reasons. First, the covariance of the spike triggered ensemble needs to be reliably estimated (with quadratically many degrees of freedom as there are stimulus dimensions compared to a linear number of degrees of freedom for the M^2^-MPH). Second, the linear filters uncovered by STC are orthogonal [1], whereas the M^2^-MPH is not constrained in this way.

It is possible to show that the M^2^-MPH firing rate 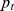 to a stimulus is given by
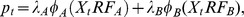
where 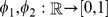 are two non-linearities and 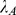 and 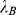 are the prior probabilities of hidden states 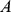 and 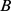, respectively. The STA model, on the other hand, is bound to model firing rates as
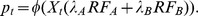



An extreme example that illustrates the failure of RF estimation with STAs is a neural response model that pools over two filters 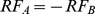 and 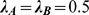. In that case the estimated RF using STA is uniform and has no predictive power at all, unlike the MPH (e.g. Fig. 5C, rotation angle 180°). A less extreme but potentially more relevant case is that of complex cells in primary visual cortex with overlapping excitatory and inhibitory oriented receptive subfields (such cells are often modeled by pooling over four oriented filters that are phase shifted 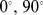, 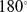, and 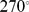, respectively [29]–[31]). A switching M^4^-MPH with four M-states can recover these phase shifted filters, whereas STA yields only a blurred RF.

### Application to Songbird Spike Data

**Figure 6:**
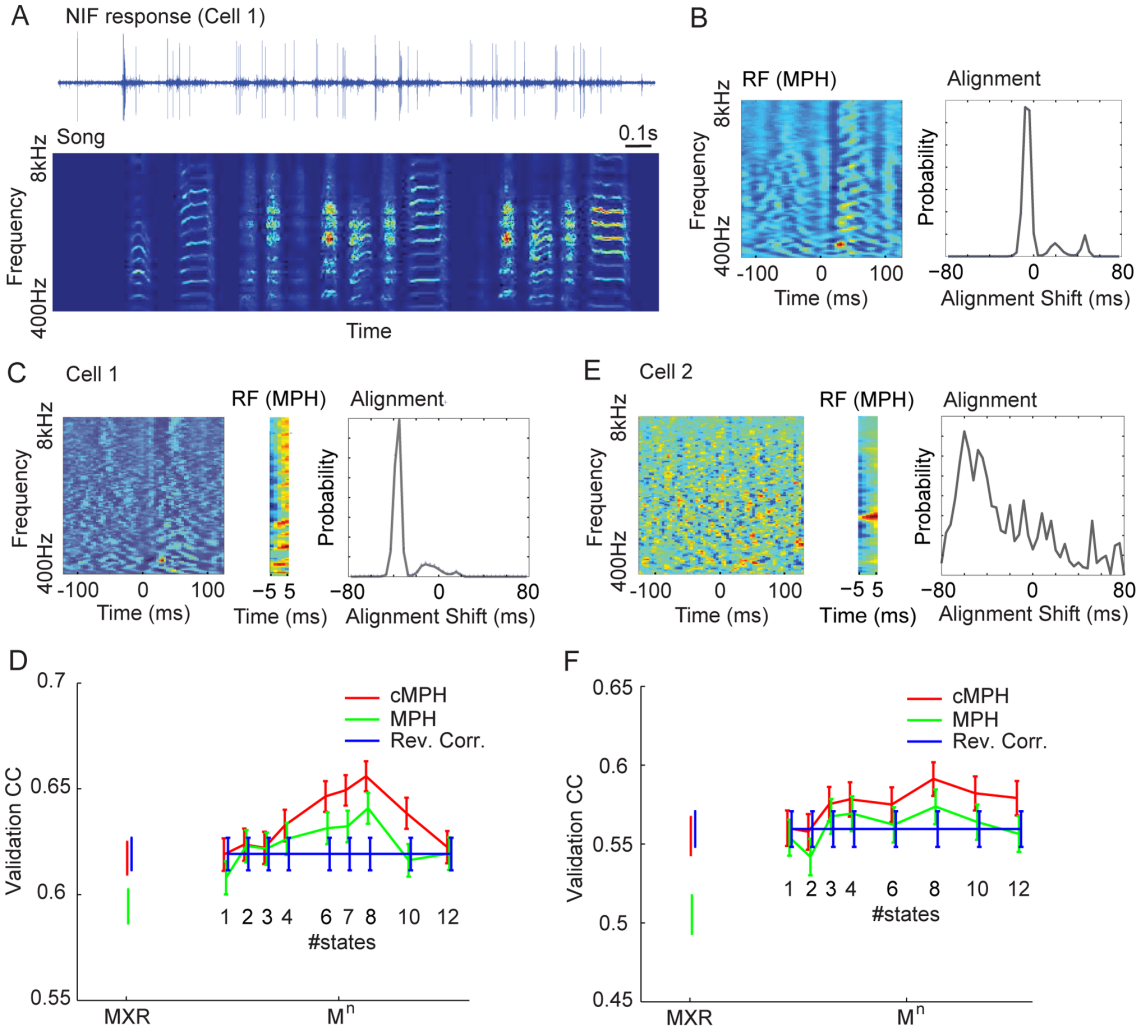
The MXR- and M^n^-MPH applied to single-unit activity in NIF of a singing zebra finch. (A) Raw extracellular voltage trace time-aligned to a log-power sound spectrogram of a zebra finch song (high sound amplitudes in red and low amplitudes in blue). (B) The MXR-MPH's RF estimate (left, high and low sound amplitudes in red and blue respectively). The red blob at about +30 ms is an indication that this cell is premotor. The width of the window is ∼0.25 s. The MXR-MPH's alignment kernel (right) is concentrated near −10 ms, yielding a total lead of NIf spikes on song of about 40 ms. (C) The RF estimated with reverse correlation (left) is similar to the MXR-MPH's RF. Middle: RF and jitter kernel of an MXR-MPH with much narrower RF window (about 10 ms wide). The total dimension of the RF is 605 (5 columns times 121 rows). Because the RF is so narrow, the spike latency is now clearly reflected in the alignment kernel (right), centered around a negative alignment shift of about 40 ms, implying that the model aligns spikes to portions of the song that occur about 40 ms after the spike. Hence, the alignment kernel strongly suggests a premotor function of this cell. (D) Predictions (5-fold cross validation) of the MXR-MPH (left, red bar) are similar to reverse correlation (blue bar). Using the non cascaded version (green bar) yields a slight drop in performance. An M^n^-MPH yields a modest improvement in prediction performance (right, peaking at 8 states) in both the cascaded (cMPH) and non-cascaded forms (MPH, error bars depict 95% confidence intervals). (E) Results for a different data set (a different cell producing 1659 spikes during about 54 s of song data containing about 60 song motifs). The RF estimated using RC reveals diffuse spectrotemporal tuning, making it nearly impossible to decide whether this cell is sensory or motor in function. By contrast, the MPH alignment kernel (right) quite clearly reveals a motor function in this cell, evidenced by the predominance of negative alignment shifts. Also, the MPH RF shows a rather narrow frequency tuning near 2.6 kHz (middle). (F) The MXR-MPH firing-rate predictions for this cell were comparable to reverse correlation predictions; M^n^-MPHs again yield a modest improvement in prediction performance.

To demonstrate that MPHs work well in practice even when the amount of available data is small and the true spike generating process is unknown, we apply the MXR- and the M^n^-MPH to extracellular spike data recorded in the forebrain nucleus interface of the nidopallium (NIf) of songbirds (Fig. 6A). NIf is a higher-order song-control nucleus; lesion and inactivation studies have shown that NIf exhibits both sensory and motor functions [32]–[35]. Multi-unit NIf activity is generally strongest shortly before and during syllable production and weakest during the times corresponding to silent intervals between syllables [36], [37]. These findings suggest a pre-vocal role of NIf spikes during song, prompting us to expect in singing birds a negative latency of NIf spikes relative to song (spikes precede sounds, as opposed to a positive latency that would result if NIf firing was sensory during vocal production). Due to the difficulty of recording in singing animals, available spike trains are relatively short (the average total spike train duration was 73 s per cell) and contain few spikes (∼1500 spikes per cell).

To investigate latencies of NIf single-unit spikes relative to song, we first fitted an LNP model using reverse correlation (RC, Fig. 6C, left). To overcome problems of over-fitting (due to the limited amount of data available) we used a regularized version of the stimulus covariance matrix:
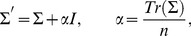
(7)where 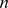 denotes the number of stimulus dimensions, 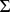 denotes the unregularized stimulus covariance matrix and 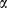 denotes its normalized trace (such regularization yielded better generalization performance). Next, we trained a *MXR-MPH* on large 0.25 s song spectrogram windows (with covariance matrices in M and X states fixed to the regularized stimulus covariance matrix 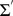 in Eq. 7). The MPH RF was similar to the reverse correlation RF (Fig. 6B), but it reflected more clearly that the cell fired before sounds and not thereafter (consider for example the stronger inhibitory band near 10 ms). MPH and reverse correlation encoding performances on a test set were comparable (Fig. 6D, left data points). Note that by construction, differences between MXR-MPH and reverse correlation RFs arise from spike-time-jitter.

To characterize response latencies (and jitter) we estimated probability distributions of the temporal offset 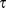 between stimulus and response in M-states via the *alignment kernel* (Fig. 6B, left). Negative lags in the alignment kernel imply that spikes occur before corresponding events in the stimulus, whereas positive lags imply that spikes occur thereafter. The alignment kernel was centered at a small negative time lag and exhibited a small temporal spread, revealing high temporal precision of NIf spike trains. Predicted responses (5-fold cross validation) for the reverse correlation model and the MXR-MPH were equally good (Fig. 6D, left data points), confirming high temporal precision of NIf spike trains.

The MPH allowed us to strongly reduce model complexity by shrinking linear filters (RF sizes) down to less than 30 ms. For such short RFs, the cell latency is reflected entirely in the alignment kernel. Based on the RF estimate in Fig. 6B and 6C, we expected the jitter kernel to be centered near −30 to −40 ms. Indeed, the kernel peaked near −40 ms (Fig. 6C, right), implying that the MPH aligned spikes to portions of the stimulus occuring about 40 ms after the spike, suggesting a premotor function of this cell and thus agreeing with the hypothesized premotor function of NIf.

Additionally we trained an M^n^-MPH with various numbers of states on the same NIf cell (unlike for the M^2^-MPH applied in the section on switching dynamics we did not constrain the means). The M^n^-MPH showed modest improvements over reverse correlation, its peak validation CC occurred at 8 states (Fig. 6D), suggesting that this NIf cell fires prior to several distinct song features.

We also analyzed data for another recording site in NIf, composed of 54 s of singing with concurrent spiking (1659 spikes, about 60 stereotyped song motifs). The RF estimated using reverse correlation (Fig 6E, left) revealed diffuse spectrotemporal tuning, making it difficult to decide whether this cell is sensory or motor in function. By contrast, the MPH alignment kernel (Fig. 6E, right) quite clearly revealed a motor function in this cell, evidenced by the predominance of negative alignment shifts. The MPH RF showed a rather narrow frequency tuning near 2,6 kHz (Fig. 6E, middle). Encoding performance for the MXR-MPH with large RF was again similar to reverse correlation (Fig. 6H, left data points), yet an M^n^-MPH yielded slightly superior performance (Fig. 6H, right data points).

## Discussion

We introduced a novel technique for neural response modeling and receptive field estimation that overcomes limitations of fixed stimulus-response relationships. We proposed to view neural coding as an alignment problem that can be tackled by mixed pair hidden Markov models (MPHs), which jointly model stimulus and response and can naturally account for noisy or systematically varying latencies as well as for context dependent neural codes that depend on internal (hidden) states. Discrete pair HMMs have been used in the context of gene alignment to find corresponding parts in related gene sequences [38]. To our knowledge they have not yet been applied to response modeling.

We demonstrated that simple MPHs with Gaussian stimulus models and a fixed shared covariance matrix are equivalent to one dimensional LNP models with sigmoid non-linearity and we extended these basic MPHs to allow flexible timing and context dependency. Thereby MPHs endow standard RF estimation techniques such as spike triggered averaging (STA) and reverse correlation with *flexible timing* and *context dependency*. We tested our approach on simulated and real data and demonstrated the benefits of alignment in terms of improved predictability of simulated and real neural responses, improved receptive field estimates as well as the capability of estimating jitter latency statistics and switching states.

Key properties of MPHs are: 1) X- and R-states that model stimulus or response alone and allow for *flexible timing* via dynamic temporal alignment, and 2) M-states that allow for *context dependency* via model switching. Using our estimation techniques, these three types of states can be freely combined in a highly flexible approach to neural coding and decoding without the need to develop additional algorithms.

We derived MPH parameters estimation updates for Gaussian mixture models with unrestricted covariance matrices (Materials and Methods). The (non-mixture) Gaussian MPHs we studied performed well in simulations (including natural stimuli), even though the assumption of Gaussian stimulus models can be violated by natural stimuli [24], [39]. In these cases, mixtures of Gaussians can be useful to approximate arbitrary stimulus distributions and overcome problems of receptive field biases [27]. Beyond mixtures of Gaussians, EM update equations for other mixture families are known as well [40] and could be adapted to MPHs.

Other modeling approaches have been pursued to estimate neural responses in the presence of spike time jitter [15], [16], [41]. One approach is to simultaneously estimate the jitter distribution and the RF using the EM algorithm [16]. This technique has been successfully applied to white noise stimuli (identity covariance) [16], but not to stimuli with non-identity covariance, i.e. natural stimuli. Furthermore, in [16] the jitter of adjacent spikes is assumed to be independent – an assumption that might be violated in cases where jitter depends on slowly varying internal states or is correlated for other reasons. The dynamic alignment technique we present here generalizes these approaches in two ways. First, in MPHs there is no need to constrain the stimulus covariance matrix, so that natural stimuli can be readily processed. Second, MPHs can account for correlated as well as uncorrelated jitter among adjacent or nearby spikes and thus allow modeling of both systematically and slowly varying spike latencies. Furthermore, in [16] the jitter distribution is explicitly assumed to be of Gaussian form whereas the jitter distribution of the MPH is implicit in the transition probabilities and has degrees of freedom commensurate with the number of hidden states and their transitions.

The ability of MPHs to emulate switching models is particularly useful given that switching dynamics are important in many neural systems. A number of other approaches have been introduced to handle response switching and context dependency. Several of them are based on hidden Markov models [42]–[48]. The hidden states in these models typically reflect neural activity but not the stimulus. Models with hidden states that reflect both stimulus and response, such as switching Kalman filters [49] or generalized linear models with hidden states [50], [51], have also been proposed. These models are similar to MPHs with only M-states but no X- and R-states. Furthermore, our approach extends these models in that stimulus-response relationships within each hidden state can be quadratic (single Gaussians, unconstrained covariance matrices) or formed by Gaussian mixtures. Another way of modeling context dependencies are “multi-linear” models encompassing a multiplicative context term (by itself modeled through a “multi-linear” model) that depends on the projection of the stimulus (in some time window) onto a set of basis functions [52]. MPHs complement such approaches by allowing more complex types of contextual influence via the underlying Markov structure. This is also an advantage over techniques like spike triggered covariance that can recover multiple filters [1], [6], [53]–[55] but cannot attribute Markovian dynamics to the individual filters. For instance, MPHs allow for context effects over very long time scales, context effects depending on hidden neural states such as up and down states (in this case MPHs also allow to infer the up and down states, for instance through the generalized Viterbi algorithm), and left-to-right HMMs [40] can incorporate behavioral context in stereotyped motor actions such as birdsong.

MPHs can bridge between data analysis and theories of neural function. Some theories of cortical function assume discrete modules of computation and representation [56], [57], for example synfire chains [47], [58] or, more generally, cell assemblies. In these theories, the role of neural activity does not only depend on the identity of the neuron but also on the (hidden) identity of modules the neuron belongs to at a certain time.

The MPHs we developed to align stimulus and neural response are based on stimuli represented with continuous probability densities and neural activity represented with discrete probabilities. It is noteworthy that both fully continuous pair HMMs that align two continuous sequences and fully discrete pair HMMs also have interesting applications. For instance, we have shown previously that a fully continuous pair HMM can be used to align the songs of a juvenile bird to the song of the bird's tutor in order to identify the parts of the song that were copied and the locations where insertions were made [59]. We have also demonstrated how fully discrete pair HMMs can be used to align spike trains [59]: by learning a discrete pair HMM on pairs of related spike trains, we obtained a “distance” measure between spike-trains, thereby generalizing state of the art spike train metrics [60].

MPHs are useful for both neural encoding and decoding. We presented algorithms for inferring neural responses and their probabilities given the stimulus (encoding). However, by symmetry of MPHs, the inference algorithms we designed can in principle be “inverted” to estimate the stimulus given neural activity (decoding) so that decoding and encoding of brain activity essentially have become the same problem.

MPHs are based on classical hidden Markov models and learning and inference algorithms other than the EM algorithm are readily available. For instance, for model parameter estimation we could have used (much faster) Viterbi training [38] or we could have optimized criteria other than data likelihood [61]. Also, there exists a large variety of very powerful analytical and computational tools developed for classical hidden Markov models that can be adapted to MPHs [61]–[63].

We will make a code package for fitting MPHs available through our website (www.ini.ch/~skollmor).

## Materials and Methods

### Ethics Statement

All experiments were carried out in accordance with protocols approved by the Veterinary Office of the Canton of Zurich, Switzerland.

### Short Introduction to Hidden Markov Models

We provide a short introduction to “normal” hidden Markov models and the associated terminology for readers unfamiliar with them.

Consider two dies at a game of chance, one with equal probabilities for its six faces (fair die) and the other with unequal probabilities (loaded die). Suppose that their holders can exchange dies for one another without you knowing. Suppose furthermore that these die switches occur randomly. All you observe is the sequence of faces without knowing whether the fair or loaded die is in place: the identity of the die is hidden from you. Hidden Markov models (HMMs) account for exactly these kinds of situations involving hidden variables. In the die example we can use an HMM with two states, *L* (loaded) and *F* (fair), for the two dies. At any point in time the HMM is in one of the two *states* corresponding to the die that is in use. Associated with each of the two states are the probabilities for the different faces to come up. These *emission probabilities* are unknown and can be learned from observations (the distribution is uniform for the fair die and non-uniform for the loaded one). Transitions between states (dies) are governed by unknown *transitions probabilities* that model how likely the die holders switch dies at any time. For two states, transitions are modeled by an unknown 2 by 2 *transition matrix* that can also be learned from observations.

An HMM can produce observations by randomly choosing transitions (die switches) and *observations* or *emissions* (faces that come up) which results in an *observation sequence* and an underlying *hidden state sequence*. However, HMMs are so useful because they can be applied in reverse: given an observation sequence, we can estimate good parameters (emission and transition probabilities) for the underlying HMM as well as the underlying hidden state sequence, which we never directly observed.

In a classical HMM (applied to stimulus-response modeling) the time lag between the stimulus and the response is fixed and together stimulus and response probabilistically depend on some hidden (non-observed) variable with Markov dynamics.

In an MPH, spike and stimuli also probabilistically depend on some hidden variable, but rather than being paired at a fixed time lag, spike and stimulus pairing is dynamic, governed by a probabilistic process. Note that MPHs are different from factorial hidden Markov models which employ a distributed state representation but model a single (possibly multidimensional) observation sequence [64].

### Formal Definition of the MPH

In the following, we present a precise definition of the MPH architecture and its learning and inference algorithms.

We denote the stimulus sequence by 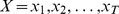 and the spiking response by 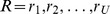 where 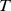 and 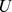 are their respective durations (typically 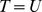). 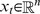 are real vectors (e.g. sound spectrograms) and 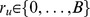 are integers (e.g., number of spikes, typically 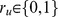 in small time bins with zero or one spike). We denote a position in the combined stimulus-response alignment matrix as 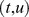, Fig. 2c. The model has three types of hidden states: X-states, which model only the stimulus, R-States which model only the response, and M-States which jointly model stimulus and response (Fig. 2c). We denote the sets of these states by 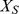, 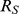, and 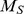. Additionally we define the union of states 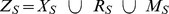. We denote sequences of hidden states by 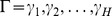 with 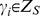 and use the notation 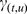 to refer to the hidden state occupied at sequence position 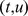. Note that in general 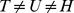 because all of stimulus, response, and hidden state sequences may be of different length. The parameters of the MPH are defined in the following.


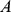
: Matrix of transition probabilities. 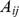 denotes the probability of transiting from hidden state 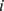 to hidden state 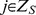



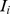
: Initial probability of hidden state 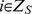



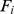
: Final probability of hidden state 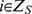



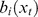
 for 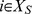: Emission probability density of the stimulus 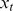 given hidden X-state 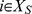



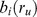
 for 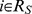: Discrete emission probability distribution of the response 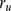 given hidden R-state 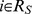



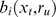
 for 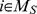: Mixed discrete-continuous emission probability of stimulus-response pair 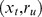 given hidden state 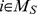


As emission probability densities associated with X and M states we use multivariate Gaussians or mixtures of Gaussians, respectively:
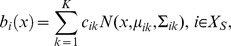
where 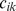 is the weight of the *k* ^th^ mixture component, 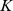 denotes the total number of mixture components (which may vary for different hidden states but this freedom is not reflected in our notation), and 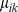 and 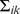 denote Gaussian mean and covariance matrix of the 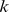
th mixture component. For M states we keep track of one such density for each possible value of 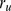 (distinct stimulus emission for each spiking state).

In the following, we define algorithms for inference in MPHs. Some of them are generalizations of well-known algorithms for normal HMMs. To infer the spiking response for a given stimulus, we derive new algorithms. In the following we denote conditional probabilities of the form 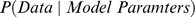 simply by 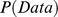, i.e., for readability we will omit the dependence on model parameters.

### Generalized Viterbi Algorithm

Assume that we have trained MPH model parameters on some data and now would like to apply the MPH to novel stimulus-response pairs. In a switching model (Fig. 2e), we would like to estimate the most likely hidden state sequence given the data to identify the switching events. In a flexible timing model (Fig. 2b) we would like to determine the optimal alignment between stimulus and response to estimate the jitter of individual spikes. In that latter case, the alignment consists of temporal offsets between stimulus and response on a moment-to-moment basis.

The generalized Viterbi algorithm for MPHs can be applied in both situations to efficiently compute the most likely hidden state sequence 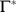 for a given stimulus-response sequence 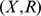:
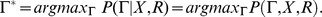



We apply an extension of the Viterbi algorithm for classical HMMs [38]. First, let 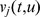 be the probability of the most likely sequence that models the stimulus up to (and including) time 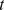, the response up to time 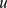, and that ends in hidden state 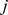. Additionally, for any state 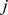 and sequence position 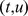, we keep track of the most likely precursor state in 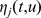. 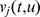 and 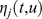 can be computed recursively (Table 1).

**Table 1:** The generalized Viterbi algorithm.

**Initialization:**		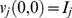
**Recursion:**	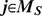 :	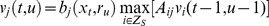
		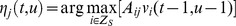
	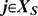 :	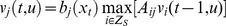
		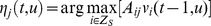
	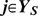 :	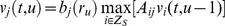
		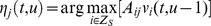
**Termination:**		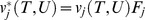

A good way to visualize the generalized Viterbi algorithm is to think of it as filling up an 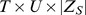 alignment tensor (Fig. 2f). The final state of the most likely hidden state sequence is then given by 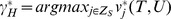 and the complete state sequence can be obtained by iteratively back-tracking the most likely precursor states 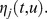


### Generalized Forward Algorithm

In many cases, we are interested in computing statistics over all possible sequences. For instance, to compute the probability 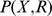 of generating a sequence pair 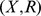 given a particular MPH (for example to compare different MPHs), we need to consider the overall probability of 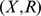 independent of the alignment. Hence we have to consider all possible hidden state sequences and not just the one with maximal likelihood. First, let 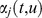 be the probability of observing the stimulus up to (and including) time 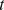, the response up to time 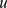, and of ending in hidden state 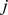. The computation of 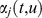 is very similar to the computation of 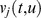, except that the max operation is replaced with a summation (to take all hidden state sequences into account, Table 2).

**Table 2:** The generalized forward algorithm.

**Initialization:**		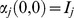
**Recursion:**	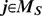 :	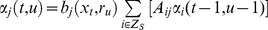
	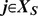 :	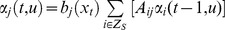
	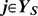 :	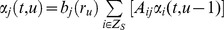
**Termination:**		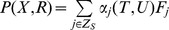

### Generalized Backward Algorithm

The backward algorithm is analogues to the forward algorithm. We present it here because it is an integral part of the EM algorithm for MPHs and the computation of posterior probabilities (see below). The backward probability 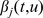 is the probability of observing the stimulus from time 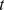 to the end and the response from time 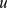 to the end (excluding 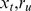), beginning at position 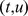 and in hidden state 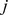 (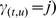. 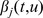 is computed recursively (Table 3).

**Table 3:** The generalized backward algorithm.

**Initialization:**		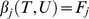
**Recursion:**	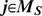 :	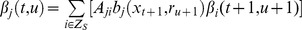
	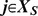	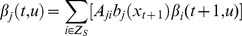
	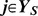	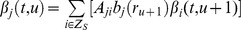
**Termination:**		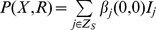

### Computing Posterior Probabilities

Assume we have trained an MPH on some data and want to determine the probability distribution over hidden states for a given stimulus-response pair and sequence position 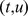. Building on the definitions of 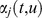 and 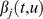 (Table 2 and 3), the posterior probability 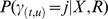 of being in hidden state 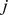 at sequence position 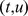 given sequence-pair 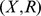 can be expressed in terms of forward and backward probabilities:
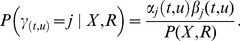
(8)


### Computing Alignment Kernels

Intuitively, the alignment kernel 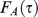 is a histogram of spike shifts over all possible state paths weighted by their respective probability.
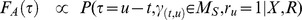



The alignment kernel is easily computed using posterior probabilities (Eq. 8) in M- and R-States at all sequence positions 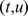 which fulfill 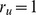:
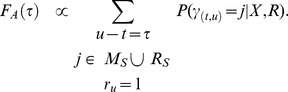



Negative lags 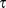 in the alignment kernel imply that spikes occur before corresponding events in the stimulus, whereas positive lags imply that spikes occur thereafter.

### Learning Model Parameters

To train an MPH on a set of stimulus-response pairs, we apply a generalization of the EM algorithm. That algorithm is analogous to its normal HMM counterpart [38], [40]. In the expectation step, the 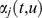 and 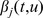 (Table 2 and 3) are used to compute the probability of each state at each sequence position, as well as the expected number of transitions between hidden state pairs. The model parameters are then re-estimated in such a way as to locally maximize the likelihood of the stimulus-response pair. For simplicity of notation we define 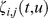 as the probability of transiting from state 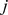 to state 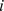 at sequence position (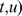:
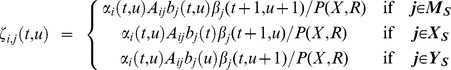



Based on 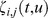, the new transition probabilities are given by
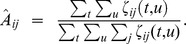



Initial probabilities are updated similarly:
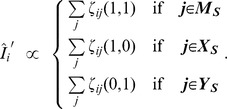



The new discrete emission probabilities for R-States are given by
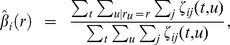
where 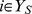 and 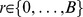.

The update of emission density parameters for X states depends on the type of continuous probability distribution used. For Gaussian mixtures with 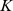 mixture components, new means and covariance matrices for the components can be computed as follows. For simplicity, we first define 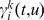, where 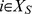 and 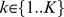:
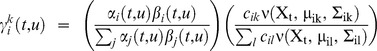



The updated mixture weights, 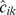, the means, 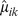, and the covariance matrices 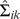 are then computed as follows:
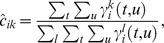


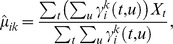


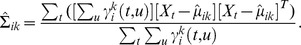



The updates for M-states are analogous. To compute the updated parameters of the mixture associated with 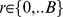 (where 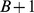 is the number of possible neural responses, i.e. 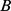 is the maximum number of spikes per time bin), we sum only over those sequence positions 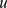 that fulfill 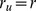.

### Most Likely Pair of Response and Hidden State Sequences

Given an MPH that was trained on some stimulus-response pairs, we can predict spiking responses to novel stimuli. This is known as encoding. Conversely, we can reconstruct stimuli from spiking responses, known as decoding. In the following, we derive two encoding algorithms for MPHs. First, we show how to compute the most likely pair of hidden-state and neural response sequences, 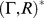, for a given stimulus 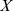. This algorithm is an extension of the generalized Viterbi algorithm (Table 1). We only present the algorithm for encoding. By symmetry, a decoding algorithm can be derived analogously.

Let again 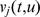 be the probability of the most likely hidden state sequence that models the stimulus up to time 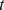 and the response up to time 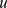 and ends in state 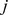. We want to compute a neural response 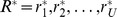 such that 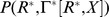 is maximized, where 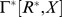 denotes the most likely state path for the sequence pair 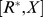 (Table 1). This is accomplished by always choosing the instantaneous neural response 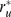, 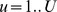 such that it maximizes the emission probability in the recursion equations (Table 4).

**Table 4:** Extended Viterbi algorithm to compute most likely pair of hidden state and neural response sequences for a given stimulus.

**Initialization:**		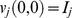
**Recursion:**	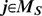 :	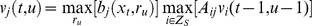
		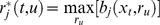
	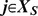 :	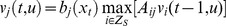
	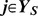 :	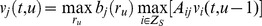
		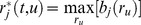
**Termination:**		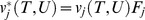

As in the generalized Viterbi algorithm (Table 1), we keep track of the most likely precursor states in 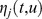. Additionally, we store the emissions that maximize the first factor on the right hand side of the recursion equations as 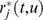. We recover the most likely pair of hidden state sequence and neural response by considering the 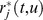 associated with the most likely state at that position (we assume that 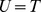; generalization to unknown 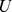 is possible, but irrelevant for our purposes).

This encoding strategy yields a spike train which depends on the most likely hidden state sequence. Such dependence can be a problem if many pairs of hidden state sequences exist with similarly high probability. Also, another caveat is that this algorithm does not provide spiking probabilities. Ideally, we would like to account for all possible hidden state sequences and compute an overall spiking or response probability for each point in time. Such improvement can be done through an extension of the forward algorithm, presented next.

### Computing the Response Probability Distribution as a Function of Time

Here we compute the probability distribution 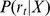 of the response 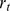 at time 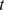 given a stimulus sequence 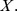 We can retrieve this probability as a posterior (using Eq. 8) after rewriting our model in the following way.

1. Replace each M-State by X- and R-states. If the response is encoded using the two symbols 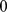 and 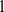 (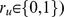, an M-State is replaced by two X-states, 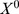 and 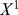, representing 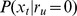 and 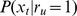 respectively and two R-states: 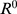 which never generates a spike, and 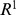 which always generates a spike. 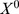 is connected to 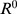 and 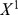 is connected to 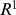 (with probability 1 in both cases). Each connection onto the former M-state is now replaced by a pair of connections to 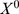 and 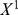, with transition probabilities each given by the product of the original transition probability and the marginal probability of a non-spike (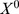) or spike (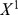) (computed by integrating the emission density of the M-state). By construction the model that results from applying this step is equivalent to the original model as far as inference is concerned.
2. Replace each of the R states in the model (except those that have been generated in step 1) by two R-states: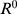 that never emits a spike and 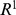 that always emits a spike. As in Step 1, the probability of spiking is encoded in the new transitions onto 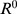 and 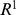
_._ By construction, the resulting model is equivalent as far as inference is concerned.

With this reformulation, we can now easily express 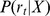 using sums over posterior probabilities of the 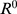 and 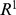 states:
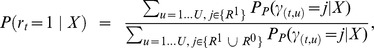
where 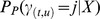 denotes the posterior probability of hidden state 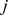 in the rewritten model, 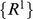 and 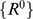 denote the sets of all ‘spiking’ and non-spiking R-states, respectively. Note that by construction 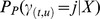 is independent of the response 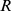. In this paper we always use this algorithm for inferring spiking probabilities in MPHs.

#### Cascaded MPHs

Inspired by [26] and the standard practice of forming model cascades in neural response modeling [1], [27], [28], we cascade the MPH, forming an NNP (non-linear-non-linear) model. For the M-MPH (section on LNP equivalence), we can realize arbitrary LNP non-linearities 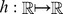. Given 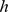 we define a mapping 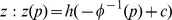 operating on the posterior spike probability 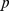 in Eq. 4. Applying this mapping 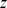 to Eq. 4 yields the desired spike probability 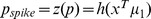.

Alternatively, we can estimate the optimal mapping 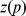 that yields the nonlinearity 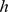 that best describes the data. We estimate this mapping from the data using the conditional probability
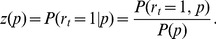
(9)


Thus, the optimal (discretized) mapping 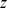 corresponds to point-wise division of two histograms, the histogram of posterior spiking probabilities given an actual spike in the numerator and the histogram of all posterior spiking probabilities in the denominator (see also [1]).

In practice, we first estimate the MPH parameters and then re-estimate the non-linearity via the mapping 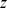 in (Eq. 9). When applying this cascaded MPH, we first compute the posterior spiking probabilities and then remap these using 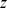. These response predictions are bound to give better results on the training set and will also improve validation performance (unless the mapping 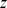 is over-fitted).

### Computational Complexity and Optimization of the Algorithms

Filling out the alignment tensor used to compute forward and backward probabilities (Fig. 2f) in a fully connected model requires 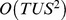 computations and additional 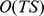 computations for emission probabilities in M and X states (as before, 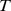 denotes the length of the 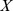 sequence (stimulus), 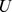 the length of the 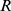 sequence (response); and 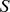 is the number of hidden states). We usually reduce this complexity by limiting the allowed temporal offset between stimulus and response to a maximal lag set by a parameter 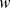. In that case, we compute only the part of the alignment tensor within a band of width 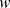 around the diagonal. Hence, the complexity reduces to 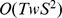. In the EM algorithm, the computational complexity is 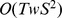.

The MPHs we studied had mostly constrained parameters, in particular constrained covariance matrices and means. We have found that free covariance matrices tend to make the models prone to over fitting and slow down training as more iterations of the EM algorithm are required (for instance, the M-MPH discussed in the section on LNP equivalence reaches the optimum in one iteration. Using free covariance matrices, convergence is gradual and it takes many more steps for the likelihood change to drop below a predefined threshold).

The EM algorithm only converges to local optima; we found that this problem can be alleviated by running the training several times from different initializations (compare Fig. 5D and the accompanying text).

### Subjects and Electrophysiology

All experiments were carried out in accordance with protocols approved by the Veterinary Office of the Canton of Zurich, Switzerland. Data were collected from juvenile male zebra finches (60–92 days old). The electrophysiological procedures are explained in detail elsewhere [65]. Briefly, microdrives were implanted using methods previously described [65]. After each experiment, the brain was removed for histological examination of unstained slices to verify the location of reference lesions. Cells were recorded during singing. During recording sessions, birds were housed in a sound isolation chamber equipped with a microphone. Extracellular voltage traces were digitized at 33 kHz and recorded for offline spike sorting.
